# Effect of C-Type Natriuretic Peptide (CNP) on Spermatozoa Maturation in Adult Rat Epididymis

**DOI:** 10.3390/cimb45020108

**Published:** 2023-02-16

**Authors:** Hu Zhao, Yuejin Yu, Chunlei Mei, Tianyu Zhang, Yafei Kang, Na Li, Donghui Huang

**Affiliations:** 1 Institute of Reproductive Health, Tongji Medical College, Huazhong University of Science and Technology, Wuhan 430030, China; zhaohu@hust.edu.cn (H.Z.); hust_yuyuejin@163.com (Y.Y.); meichunlei_1993@126.com (C.M.); tianyuzhang_tongji@163.com (T.Z.); kangyafeitt@163.com (Y.K.); alina173@163.com (N.L.); 2 Department of Human Anatomy, Tongji Medical College, Huazhong University of Science and Technology, Wuhan 430030, China; 3 Shenzhen Huazhong University of Science and Technology Research Institute, Shenzhen 518063, China

**Keywords:** C-type natriuretic peptide, NPR-B, testosterone, epididymal sperm maturation, acquisition of sperm motility

## Abstract

C-type natriuretic peptide (CNP) is highly expressed in male reproductive tissues, such as the epididymis. The aim of this study is to explore the role of CNP in the maturation of rat epididymal spermatozoa. First, the expression levels of CNP and its specific natriuretic peptide receptor-B (NPR-B) were detected in various tissues of rats and epididymis at different stages after birth. Then a castrated rat model was established to analyze the relationship between testosterone and CNP/NPR-B expression in the epididymis. Finally, CNP and different inhibitors (NPR-B inhibitors, cGMP inhibitors) were used to incubate epididymal sperm in vitro to examine sperm mobility and expression of sperm maturation-related factors. The results showed *CNP/NPR-B* mRNAs were expressed in all tissues of rats, but were extremely highly expressed in male genital ducts (seminal vesicle, prostate and epididymis). The expression of *CNP/NPR-B* in epididymis was the highest at birth and the fifth week after birth. In the epididymis, CNP/NPR-B were highly expressed in the caput and located in the epididymal epithelial cells. After castration, the expression of *CNP/NPR-B* decreased sharply and was restored quickly after testosterone supplementation. In vitro, CNP could significantly promote the acquisition of epididymal sperm motility through the NPR-B/cGMP pathway and induce the expression of sperm maturation-related factors (such as Bin1b, Catsper 1, Dnah17, Fertilin). This study shows that CNP plays a role in epididymal sperm maturation. The mechanism of CNP is to promote the acquisition of epididymal sperm fluidity through the NPR-B/cGMP signaling pathway and also to regulate sperm maturation-related genes. Moreover, the expression of *CNP/NPR-B* was regulated by testosterone.

## 1. Introduction

The epididymis is a fundamental reproductive organ, responsible for sperm maturation, transportation and storage. The gametes produced from the testes are functionally immature and then acquire motility and fertilizing abilities during transportation in the epididymis [1]. The epididymis secretes vesicles and forms a protein matrix to provide a suitable environment for sperm maturation [2]. Therefore, the sperm protein, lipid, and small RNA content in the epididymis change greatly when they interact with these substances in the epididymal lumen [3]. For example, some secretory proteins such as P26h, sperm associated antigen 11, heat-shock protein 1 are deposited on the surface of sperm, while spermatozoal proteins (cyritestin, fertilin, CE9 and others) are also changed in this intraluminal milieu [4]. Furthermore, genetic alterations of epididymal genes can lead to decreased sperm motility, morphological abnormalities of spermatozoa, and subfertility [5].

C-type natriuretic peptide (CNP), the third member of the natriuretic peptide family, generates intracellular cyclic guanosine 3′,5′-monophosphate (cGMP) through the binding of its specific natriuretic peptide receptor-B (NPR-B), followed by various molecular effects [6]. CNP has been shown to play a key role in female reproduction by preventing precocious meiotic maturation [7]. In male reproduction, some studies have demonstrated that CNP was extremely highly expressed in seminal plasma and epididymis [8,9,10]. Sogawa et al. [11] showed that adult *NPR-B* (slw/slw) mice exhibited infertility with apparently normal spermatogenesis, though the mechanism was unclear. Knockdown of *NPR-B* by RNAi lead to dysfunction of mouse Leydig cells via S-phase cell cycle arrest and testosterone secretion decrease [12]. CNP could regulate expression of ABP and TRF in Sertoli cells through the NPR-B/cGMP/PKG signaling pathways [13]. Kong et al. [14] found that CNP secreted by oviductal ampulla attracted sperm accumulation in the capillary through Npr2 on the midpiece of flagellum but could not attract spermatozoa from Npr2 mutant mice. Our previous studies have shown that CNP could promote sperm motility [15], induce sperm hyperactivation and acrosome reaction by the cGMP/PKG signalling pathway, via Ca^2+^ influx and tyrosine phosphorylation [14]. Moreover, the concentration of CNP in the semen of asthenospermia patients was lower than that of healthy people. Additionally, CNP could improve sperm motility and the reproductive function of asthenozoospermia patients [16]. The results indicated that CNP is closely related to the male reproductive function. However, the significance of this especially high CNP expression in the epididymis and its role is unclear.

In combination with the high expression of CNP in the epididymis and its influence on sperm function (such as motility and capacitation), we suspect that infertility of NPR-B (−/−) male mice may be due to the maturation defect of sperm in the epididymis, which may, in turn, lead to sperm motility disability and infertility. This study will first clarify the expression level of CNP/NPR-B in various rat tissues and their location in the epididymis. Then, a castration rat model will be established to explore the relationship between CNP/NPR-B expression and testosterone. Finally, this study will investigate the effect of CNP on sperm motility and maturation in order to elucidate the important role of CNP in the process of epididymal sperm maturation.

## 2. Materials and Methods

### 2.1. Animals

Male Sprague–Dawley (SD) rats (weighing 400 ± 20 g, 10–12 W) were purchased from the animal center of Tongji Medical College (Wuhan, Hubei, China). They were raised in a 12 h dark and 12 h light cycle and were free to obtain food and water. They were treated according to the guidelines of the Animal Research Committee [17] and approved by the Centre of Experimental Animals of Huazhong University of Science and Technology (No. S1188).

### 2.2. Establishment of Castrated Rat Model

Castrated rats were modeled as previously described [18]. Briefly, testicular artery ligation and bilateral testes removal were performed on 10–12 w male SD rats. Castrated rats were divided into nine groups (four in each group). The first five groups were executed after 0, 1, 3, 5, and 7 days of castration. The latter four groups were injected with androgens (3 mg/kg/d) 10 days after castration and were executed 1, 3, 5, and 7 days after the start of androgens. RNA was extracted from epididymal tissue of each group. The expression of CNP and NPR-B in rat epididymis was detected by fluorescence quantitative PCR. Serum testosterone was detected by ELISA, similar to the detection of CNP, cGMP using ELISA.

### 2.3. Determination of the CNP/NPR-B Expression in Different Tissues of Rats by RT-PCR

RNA was extracted from heart, liver, spleen, lung, kidney, brain, testis, epididymis, prostate, and seminal vesicle glands of 10–12 w male SD rats. As previously described, two-step, real-time reverse transcription polymerase chain reaction (RT-PCR) was performed [13]. Briefly, each sample was transferred to a 1 mL Trizol reagent. One (1) µg total RNA was reverse-transcribed into first-strand cDNA using a First Strand cDNA Synthesis Kit (Thermo, Waltham, MA, USA). Then, cDNAs were used to perform the subsequent real-time fluorescence quantitative PCR with SYBR Green Master Mix (DBI, Freiberg, Germany). The relative expression of target genes was standardized by GADPH, and the final gene expression was calculated with 2^−ΔΔCT^. The primer information of all revolved genes can be seen in Table 1. The experiment was repeated three times to estimate expression stability.

### 2.4. Protein Concentration of CNP and cGMP in the Epididymal Fluid of Rats Using ELISA

Epididymal fluids at different segments in adult male SD rats were collected. The epididymal fluids containing epididymal tissue were centrifuged at 100× *g* for 2 min to remove tissue fragments. The supernatant was absorbed into a new centrifuge tube and centrifuged at 1000× *g* for 10 min to remove the sperm and unrelated cells. CNP and cGMP concentrations were quantified using a Rat Cyclic Guanosine Monophosphate (cGMP) Elisa Kit and CNP Elisa Kit (Elabscience Biotechnology Co., Wuhan, China) following the manufacturer’s instructions.

### 2.5. Immunohistochemistry

Immunohistochemistry was performed as previously described [19]. Briefly, male rats were sacrificed via neck dislocation. The epididymis was removed, fixed with 4% paraformaldehyde, and embedded in paraffin. The tissues were sliced, sectioned, dewaxed, rehydrated, and subjected to antigen retrieval in a microwave oven. Then the slices were incubated with primary rabbit polyclonal antibody against CNP (sc-20952) and goat polyclonal antibody against NPR-B (sc-16870) (Santa Cruz Biotechnology Inc., Santa Cruz, CA, USA). Negative controls used rabbit or goat non-immune serum instead of primary antibodies. After several washings with PBS, the slices were hatched with secondary antibodies (Boster Biotechnology Co., Ltd., Wuhan, China). Finally, the slices were photographed under an Olympus BX-40 microscope (Olympus Corp., Melville, NY, USA).

### 2.6. Expression of NPR-B in the Spermatozoa by Immunofluorescence

Indirect immunofluorescence was applied to analyze the distribution of NPR-B in the different segments of epididymal spermatozoa. In short, the spermatozoa were dried, fixed, infiltrated, treated with serum, and tested with anti-human NPR-B polyclonal antibody (1:200) (Santa Cruz Biotechnology Inc., Santa Cruz, CA, USA). Then, the spermatozoa were incubated with fluorescein isothiocyanate binding secondary antibody (1:100) (Servicebio Technology Co., Ltd., Wuhan, China). The negative control used 0.01 mol/L phosphate buffered saline (PBS; pH 7.4), replacing the primary antibody. Immunofluorescence results were evaluated using confocal laser scanning microscopy.

### 2.7. Mobility and Maturation of Epididymal Sperm Treated with CNP In Vitro

The SD rats were sacrificed via neck dislocation, and the epididymis was separated [15]. Then sperm were placed in HTF medium (Irvine Scientific, Santa Ana, CA, USA) with or without CNP (10^−7^ mol/L) at 37 °C. Ten (10) µL of sperm suspension was extracted to observe under the microscope; 200 sperm were counted and the percentage of forward motion (PR) and non-forward motion (NP) sperm were recorded according to reference [20]; then, sperm density was calculated by a blood cell counting plate. This process was repeated three times. Next, in order to detect sperm maturation-related factors, the sperm RNAs at the caput of epididymis were extracted to perform the RT-PCR after a 6-h incubation with CNP.

### 2.8. Detection of the Signaling Pathway of CNP in Epididymal Sperm Motility

To explore the signal pathway of CNP in rat epididymal sperm motility, different inhibitors were added to the HTF medium. The sperm suspensions were divided into six groups: control group (PBS medium only), CNP group (10^−6^ mol/L), 8-Br-cGMP group (Sigma, St. Louis, MO, USA) (10^−4^ mol/L), CNP with KT5823 group (10^−6^ mol/L CNP and 10^−4^ mol/L KT5823, 8-Br-cGMP with KT5823 group (10^−4^ mol/L 8-Br-cGMP and 10^−4^ mol/L KT5823) and CNP with NPR-B antagonist (P19) group (10^−6^ mol/L CNP and 10^−6^ mol/L P19). After 4 h of incubation, sperm motilities were detected. After a 6-h incubation, the gene expression of sperm maturation-related factors was detected.

### 2.9. Statistical Analyses

All data were statistically analyzed using SPSS software, Version 23.0 (IBM Corp., Armonk, NY, USA). The means ± standard deviations (SD) and 95% confidence intervals (CI) were reported for quantitative data, and percentages were reported for categorical data. The expression of CNP and NPR-B at different segments of epididymis and sperm motility inhibited by different inhibitors following stimulation by CNP were analyzed using general linear models—repeated measures ANOVA. The expression of maturation-related genes in the epididymal caput sperm was evaluated by one-way ANOVA. A level of *p* < 0.05 was considered statistically significant.

## 3. Results

### 3.1. Expression of CNP/NPR-B in Different Rat Tissues and in Epididymis at Various Times after Birth

**Figure 1:**
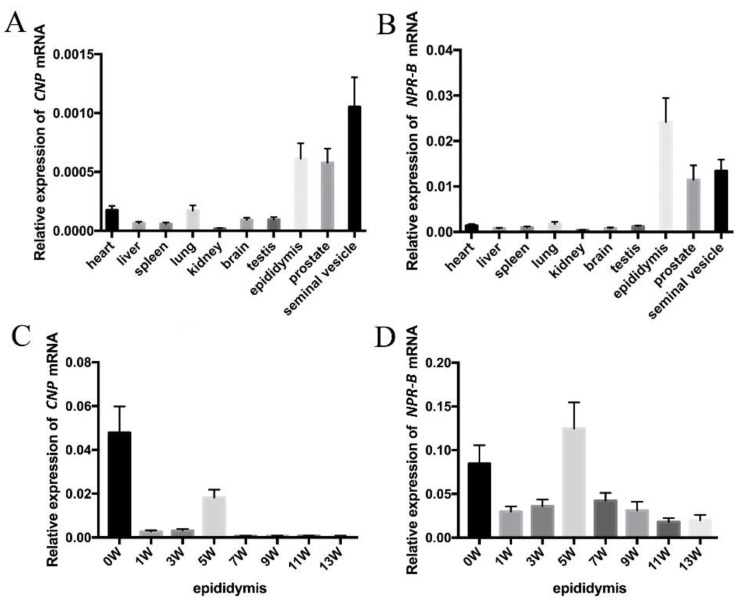
The expression of *CNP/NPR-B* in rat. (**A**,**B**) The expression of *CNP* (**A**) and *NPR-B* (**B**) mRNA in different rat tissues. (**C**,**D**) The expression of *CNP* (**C**) and *NPR-B* (**D**) mRNA in epididymis at different stages after birth.

Based on PCR analyses on different rat tissues, *CNP* and *NPR-B* mRNA are expressed in all tissues and are more highly expressed in the male genital tract (seminal vesicle, epididymis and prostate) than in other tissues (such as heart, liver, spleen, lung, kidney, brain and testis) (Figure 1A,B). The expressions of *CNP* mRNA in epididymis was highest at birth (0 W), decreased dramatically at 1 W and 3 W, and then increased at 5 W with additional decreases at 7 W, 9 W, 11 W and 13 W. Similarly to *CNP*, the expression of *NPR-B* also showed two peaks—one at birth (0 W) and the other at the fifth week (5 W).

### 3.2. Expression of CNP/NPR-B in Different Segments of Rat Epididymis

**Figure 2:**
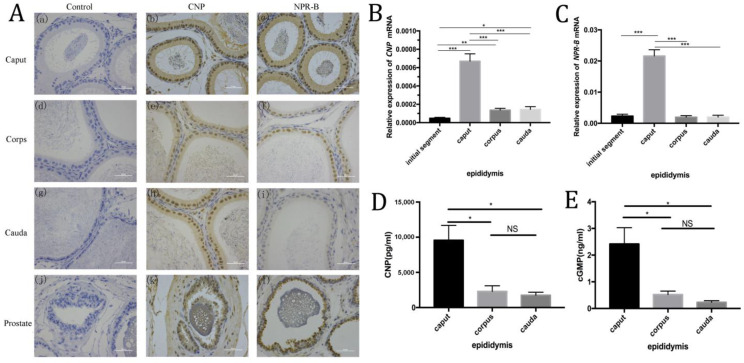
The expression of CNP/NPR-B in the different segments of epididymis. (**A**) Immunohistochemistry shows the localization of CNP and NPR-B in different segments of the epididymis and prostate. a–c: caput of epididymis; d–f: corpse of epididymis; g–i: cauda of epididymis; j–l: prostate. (**B**,**C**) The level of *CNP* (**B**) and *NPR-B* (**C**) mRNA in different segments of epididymis detected by RT- PCR. (**D**,**E**) The content of CNP (**D**) and cGMP (**E**) in different segments of epididymal fluid by ELISA. (* *p* < 0.05, ** *p* < 0.01, *** *p* < 0.01, NS: not significant).

Immunohistochemistry showed CNP and NPR-B were mainly located in rat epididymal epithelial cells (such as principal cells), and the expression of CNP and NPR-B in the caput were higher than those in the corps and the cauda (Figure 2A). RT-PCR showed similar trends, i.e., that the level of *CNP* and *NPR-B* mRNA in the caput were much higher than in other segments (such as the initial segment, corps and cauda) (*p* < 0.01) (Figure 2B,C). In rat epididymal fluid, ELISA results demonstrated that the contents of CNP and cGMP in the caput were significantly higher than those in the corps and the cauda (*p* < 0.05) (Figure 2D,E).

### 3.3. Expression of CNP, NPR-B in the Castrated Rat Model

**Figure 3:**
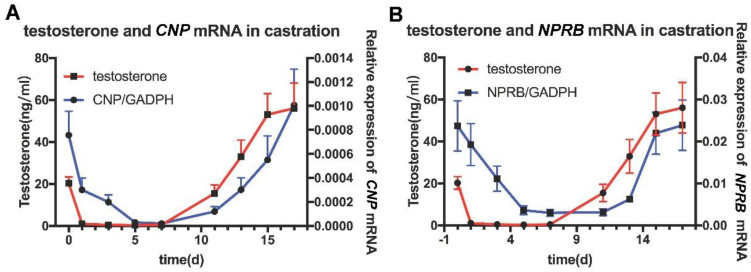
The expression of *CNP/NPR−B* in the castrated rat model. The red line represents the content of testosterone in the serum, while the blue line represents the expression of *CNP* (**A**) or *NPR−B* (**B**) mRNA in the epididymis.

In order to investigate whether *CNP/NPR-B* is regulated by testosterone, castration rat models were established. The expression of *CNP/NPR-B* mRNA in the epididymis decreased significantly on the first day (D1) after castration and almost disappeared on D5 (Figure 3). After testosterone was supplemented at D10, the expression of *CNP/NPR-B* mRNA was immediately restored and increased with the level of serum testosterone (Figure 3). The changes of CNP and NPR-B followed that of testosterone and showed the similar trends.

### 3.4. Effect of CNP on Motility of the Epididymis Sperm In Vitro

**Figure 4:**
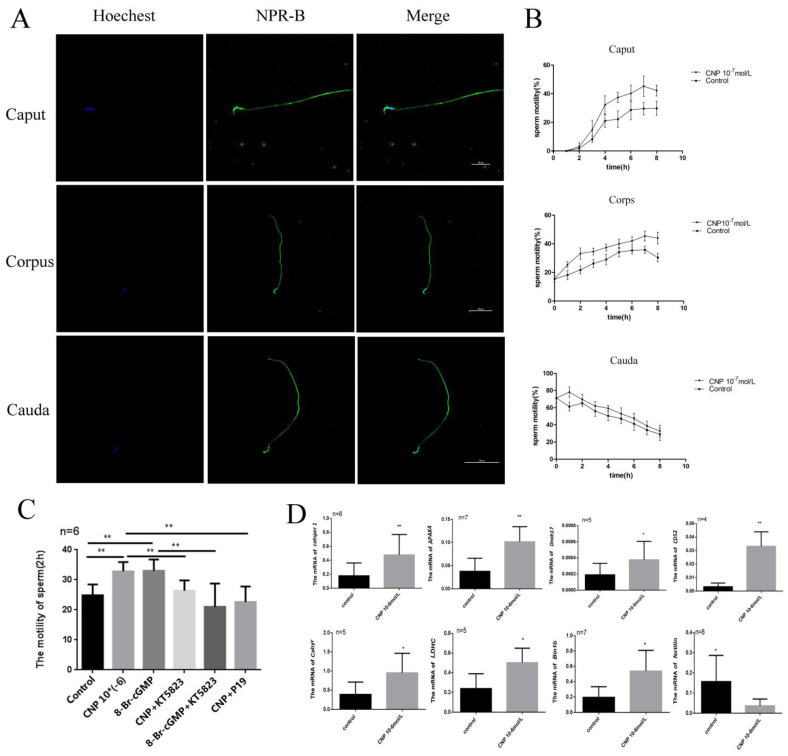
The effect of CNP on maturation of the epididymis sperm in vitro. (**A**) Spermatozoa immunofluorescence in the different segments of the epididymis detected by laser confocal photography (600×). (**B**) Sperm motilities at different segments of epididymis detected at 0, 2, 4, 6 and 8 h after incubation with CNP. (**C**) Sperm motility at the caput of the epididymis after incubation with either CNP, 8-Br-cGMP, KT5823, or NPR-B antagonist. (**D**) Expression of maturation-related genes in the epididymal caput sperm after incubation with CNP for 6 h. (* *p* < 0.05, ** *p* < 0.01).

Immunofluorescence showed that NPR-B is mainly located in the head and middle tail of spermatozoa at different segments of epididymis (Figure 4A). After incubation with CNP, the sperm motility in the caput, corps, and cauda of the epididymis all increased (Figure 4B). Interestingly, CNP could promote the acquisition of sperm motility in the caput of the epididymis (Figure 4C). To explore the signal pathway of CNP in epididymal sperm, cGMP analogues (8-Br-cGMP), a PKG inhibitor (KT5823), and an NPR-B antagonist (P19) were added (Figure 4C). The results exhibited that 8-Br-cGMP, similarly to CNP, could increase the sperm motility (*p* < 0.01) (Figure 4C). On the contrary, P19 and KT5823 could inhibit the effect of CNP on epididymal sperm motility after 2 h in vitro culture (*p* < 0.01) (Figure 4C).

### 3.5. Effect of CNP on Maturation-Related Factors of the Epididymis Sperm In Vitro

After incubation with CNP 10^−6^ nmol/L for 6 h, the gene expression of sperm maturation-related factors was detected. The results showed CNP can significantly increase the expression of *Catsper 1,* A-kinase anchor protein 4 (*AKAP4*), Cluster of Differentiation 52 (*CD52*), lactate dehydrogenase (*LDCH*), dynein axonemal heavy *chain 17 (Dnah17)* and *Bin1b* (* *p* < 0.05, ** *p* < 0.01, Figure 4D), and decrease the expression of *Fertilin* ((*p* < 0.05) (Figure 4D).

## 4. Discussion

This study first explored the role of CNP in epididymal sperm maturation. Results showed that CNP/NPR-B was extremely highly expressed in the male reproductive duct (epididymis, seminal vesicles, and prostate gland). In the epididymis, CNP/NPR-B were located mainly in the caput, and their expression was regulated by testosterone. CNP could not only initiate epididymal sperm motility through the cGMP pathway but also regulate the expression of sperm motility and mature-related genes (such as Bin1b, Catsper 1, Dnah17, and Fertilin), which indicated that CNP could regulate the process of sperm maturation in the epididymis.

The epididymis has many tissue-specific or predominant genes. For example, Lipocalin 5 (Lcn5) and Lipocalin 8 (Lcn8) are epididymis-specific lipocalin genes [21]. Robertson M et al. analyzed publicly available human and mouse RNA-seq datasets and found a plethora of novel testis- and epididymis-specific genes (Spint3, Spint4, Spint5, and Ces5a) [22]. Oh J et al. identified and characterized 32 novel epididymis-specific or predominant genes by an integrative approach and found that six of the 32 novel genes were identified as β-defensins and eight contained a protease inhibitor domain [23]. In our studies, we found CNP/NPR-B also were epididymis predominant genes, which were highly expressed in the epididymis. This result was similar to other study reports [8,9,10].

The epididymis is immature at birth, and epithelial cells acquire their fully differentiated phenotype during an extended postnatal period [24]. Moreover, the process of epithelial cell differentiation needs luminal testicular factors [25]. First, mature spermatids release from rat testes around postnatal D44 [26]. Then, the sperm undergo the maturation process in the epididymis [27]. Bin1b, which is exclusively expressed in the caput region of the rat epididymis, starts to express on the 30th day after birth and reaches its peak in the 9th month after birth, which is responsible for sperm maturation, storage, and protection [28]. Mehmet Özbek et al. showed NPPC staining was observed intensely in all epithelial cells in the rat epididymis during the postnatal period [29]. In our experiments, we detected the expression levels of CNP and NPR-B in the rat epididymis at different postnatal periods and found that the expression levels of CNP/NPR-B in the epididymis peaked at 0 W and 5 W after birth. We presumed that high expression of CNP/NPR-B at birth may be attributed to fetal epididymis development, while the peak expression at postnatal 5 W was involved in preparing proteins for the initial coming wave of sperm.

The epididymis Is mainly divided ”nto ’hree parts: caput, corps, and cauda. The caput mainly contributes to sperm maturation, while the cauda plays a role in sperm storage [30]. Each part has different gene expression profiles, which ensure different epididymis functions necessary for different stages of sperm maturation [31]. Zhao W et al. examined region-specific gene expressions in yak epididymis by RNA-seq analysis and showed that the caput segment relatively highly expressed Sal1, LCN6, PTDS, DEFB109, DEFB 119, DEFB 123, SPAG11, PROC, CST3, ADAM28, KCNJ12, and SLC13A2, while the cauda epididymis highly expressed MCT7, PAG4, OAS1, TGM3, and PRSS45 [32]. Kim SZ et al. demonstrated that specific natriuretic peptides receptors are localized in surrounding smooth muscle cells of the duct of the epididymis of the freshwater turtle, which suggests involvement in the control of the transport of sperm [33]. Mietens A et al. showed that atrial natriuretic peptide (ANP) and nitric oxide (NO) promote relaxation of smooth muscle cells in the epididymal duct to ensure that immotile spermatozoa acquire their fertilizing capacity [34]. Thong A et al. found that NPR-B was located in epididymal epithelial cells, while neutral endopeptidase (NEP) lay exclusively in apical parts of epithelial cells and that NEP inhibitors could raise cGMP by decreasing CNP degradation while being involved in the regulation of epididymal function [35]. In this experiment, the expression of CNP/NPR-B in different segments of the epididymis were examined, and the results showed that both CNP and NPR-B were highly expressed in the caput of epididymis and located in the epididymal epithelial cells, which indicated CNP/NPR-B might be involved in sperm maturation in the epididymis.

Androgen is essential for the maturation of spermatozoa in the epididymis [36]. Half of the epididymis-specific or -predominant genes are regulated by androgens, and most of the androgen-regulated genes were located in the caput region [23,37]. SPAG11A, a member of the beta defensin protein family, was expressed exclusively in the principal cells of the mouse caput epididymis and regulated by androgen—typical of genes that are involved in creating a suitable microenvironment for sperm maturation [38]. The gene expression of RNase9, a potential regulator of sperm maturation in the rat epididymis caput, decreased dramatically after castration and was restored with androgen replacement, which exhibited an androgen-dependent expression pattern [39]. Fernandez CD et al. [40] found that DES accelerated sperm transit time in the epididymis and consequently decreased sperm density and diminished sperm motility; testosterone supplementation was able to restore the transit time to values close to normality and improved the sperm motility. Khurana ML. et al. [41] showed that different natriuretic peptides (including CNP) could stimulate steroidogenesis in purified mouse Leydig cells. Dehydroepiandrosterone (DHEA) treatment caused high expression of CNP and NPR-B in granulosa cells, which is involved in the oocyte meiotic arrest and very low ovulation rate in the polycystic ovary syndrome [42]. The amino-terminal propeptide of CNP levels increases markedly during testosterone treatment in children of short stature due to GH deficiency, known as idiopathic short stature [43]. In this study, CNP/NPR-B mRNA expression in the rat epididymis dramatically decreased after castration and was restored quickly after added testosterone in vivo, suggesting that CNP/NPR-B expression was regulated by testosterone. Epididymal sperm maturation mainly includes two aspects—the acquisition of motility and the gain of fertilization ability. This process happens mainly in the caput of the epididymis. As the spermatozoa moved through the corpus epididymis, motility increased sharply, and continued to improve through the cauda epididymis and vas deferens [44,45]. Björkgren et al. demonstrated that mouse beta-defensin 41 (DEFB41) specifically expressed in the caput of the epididymis and could initiate sperm motility [46]. Bin1b participates in the initiation of epididymal sperm motility along with antibacterial activity [28]. Deficient human Bin1b will lead to sperm vitality decline and genital tract infection [47]. Sperm motility initiation in the epididymis can be induced by changes in metabolism, cAMP (cyclic adenosine mono-phosphate), calcium and pH, and some protein kinases and phosphatases (such as sperm specific protein phosphatase PP1γ2, glycogen synthase kinase 3, and the calcium-regulated phosphatase calcineurin) are involved in epididymal sperm maturation [48]. This experiment showed CNP could initiate epididymal sperm motility, and its mechanism was enacted via stimulating the cGMP pathway by binding NPR-B in sperm. These findings are in line with those of our previous study [16].

The acquisition of fertilization ability in epididymal sperm is mainly manifested in the changes of lipid and protein on the sperm surface [49,50]. This process is controlled by epididymal epithelial cells that would respond to their surrounding environment and communicate with spermatozoa [50]. Zhao et al. demonstrated that the rat epididymis-specific β-defensin 15 (Defb15) can bind to the acrosomal region of caput sperm and that inhibiting the function of Defb15 resulted in a significant decline in sperm motility and embryonic development failure [51]. Fertilin is a kind of sperm plasma membrane protein that can mediate sperm-egg membrane interactions [52]. Moreover, fertilin will migrate from the acrosomal region to the acrosomal ridge during sperm maturation [53]. Our studies showed that CNP, mainly located in the caput of the epididymis, could regulate the expression of maturation-related genes (such as LDCH, Dnah17, Bin1b, Catsper 1, APAK4, and CD52) in the epididymal sperm.

## 5. Conclusions

**Figure 5:**
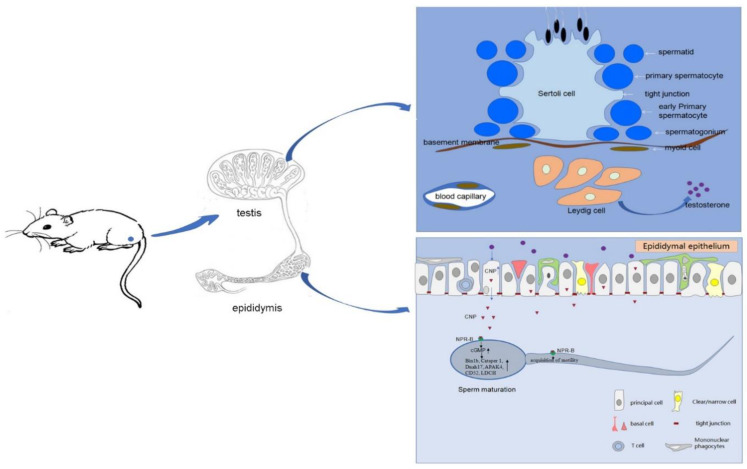
The role of CNP in sperm maturation. Testosterone secreted by Leydig cells could promote the expression of CNP/NPR-B in rat epididymal epithelium, especially in the caput. CNP not only promotes the acquisition of epididymal sperm mobility via the NPR-B/cGMP signal pathway but also stimulates sperm mature-related genes (such as Bin1b, Catsper 1, Dnah17), thus playing a role in sperm maturation in epididymis.

In summary, we detected a high expression of CNP/NPR-B in rat epididymis, especially in the caput, and found that the expression was regulated by testosterone. CNP not only promote the acquisition of epididymal sperm mobility via the NPR-B/cGMP signal pathway, but also regulate sperm mature-related genes (such as Bin1b, Catsper 1, Dnah17, and Fertilin), thus playing a role in sperm maturation in the epididymis (Figure 5). Our study highlighted the important role of CNP in epididymal sperm maturation, including promoting the acquirement of sperm motility, which may provide a potential therapy for patients with abnormal sperm maturation.

## Figures and Tables

**Table 1:** Real-time RT-PCR primers.

Gene Serial Number	Gene	Forward Primer	Reverse Primer	Length (bp)	Annealing Temperature
NM_017008.4	GADPH	5′ CAGTGCCAGCCTCGTCTCAT 3′	5′ AGGGGCCATCCACAGTCTTC 3′	202	60 °C
NM_053750.1	CNP	5′ CAACGCGCGCAAATACAAA 3′	5′ TAACATCCCAGACCGCTCATG 3′	163	60 °C
XM_006238010.4	NPR-B	5′ CCAAATTCTACCCCACTCTG 3′	5′ TAAGAAGTGTCACCACCTGC 3′	183	60 °C
XM_039101330.1	Catsper 1	5′ TTTACCTGCCTCTTCCTCTTCT 3′	5′ ACCAGGTTGAGGAAGATGAAGT 3′	227	60 °C
NM_024402.1	Akap4	5′ AGAGTCATCGCAGCATCCAA 3′	5′ TAATTCGCCGTCTGACCTGG 3′	258	60 °C
NM_053983.2	CD52	5′ AGAAAAACCCCTGGGAAACC 3′	5′ GTTGGGGTGTCTCTTGCTAC 3′	184	60 °C
NM_017266.2	LDHC	5′ TCCACCTGTGAAGGCTCAAC 3′	5′ ATGCAGAAGATCCAGTGCCTC 3′	221	60 °C
XM_039087513.1	Dnah17	5′ TGGAGGAGGTCCTCTACTCT 3′	5′ CCCATCACGAACATCTCGTT 3′	121	60 °C
NM_145087.2	Bin1b	5′ AGTCTCATCTGCTTTCCTGCAC 3′	5′ CACGGTGTTTCTGATTCCAGG 3′	128	60 °C
